# Dual Autoimmunity: Evans Syndrome and Multiple Sclerosis

**DOI:** 10.7759/cureus.113997

**Published:** 2026-08-05

**Authors:** Alexa Markl, Marisa Warren, Joshua Iskander, Camryn Warren, Sarah Faisal, Akash Parashar

**Affiliations:** 1 School of Medicine, Lake Erie College of Osteopathic Medicine, Erie, USA; 2 School of Medicine, University at Buffalo, Buffalo, USA; 3 Department of Internal Medicine, University at Buffalo, Buffalo, USA

**Keywords:** autoimmune hemolytic anemia, coombs, evans syndrome, immune thrombocytopenia, multiple sclerosis, rituximab

## Abstract

Evans syndrome (ES) is an uncommon autoimmune disorder associated with autoimmune hemolytic anemia and immune thrombocytopenia (ITP). Patients generally present with petechiae, purpura, pallor, and fatigue. Diagnostic criteria include a positive direct antiglobulin test, reticulocytosis, decreased haptoglobin, and elevated indirect bilirubin and lactate dehydrogenase levels. ES can be associated with other autoimmune conditions, including systemic lupus erythematosus, rheumatoid arthritis, and autoimmune hepatitis. However, the co-occurrence of ES and multiple sclerosis (MS) is exceedingly rare in the scientific literature. We report the case of a 46-year-old female patient with MS and a previous diagnosis of ITP. Further diagnostic workup confirmed ES. This report highlights the need to consider ES in patients with treatment-resistant ITP. Additionally, it discusses MS as a co-occurring autoimmune condition with ES that warrants further investigation within the broader spectrum of autoimmune disorders.

## Introduction

Evans syndrome (ES) is a rare autoimmune disorder characterized by cytopenia in two or more cell lines [1]. It often demonstrates the presence of autoimmune hemolytic anemia (AIHA) and immune thrombocytopenia (ITP), with thrombocytopenia typically preceding AIHA, delaying the diagnosis of ES by years. AIHA is a disorder of host immune system dysregulation involving RBC destruction, leading to the persistence of autoreactive T and B lymphocytes [2,3]. The most important diagnostic test for AIHA is the direct antiglobulin test (DAT), which uses monospecific and polyspecific anti-human globulins, anti-IgG, and anti-C3d [4]. In warm AIHA (wAIHA), DAT detects IgG antibodies, often bound to C3 on the surface of RBCs. As a result, extravascular hemolysis occurs through splenic macrophage-mediated clearance of antibody-coated erythrocytes [4]. Other diagnostic criteria for AIHA include reticulocytosis, increased indirect bilirubin and lactate dehydrogenase levels, and decreased haptoglobin [5]. ITP, also known as idiopathic thrombocytopenic purpura, is an autoimmune condition that affects both children and adults due to IgG autoantibodies against circulating platelets [6]. Patients typically present with cutaneous bleeding, such as a nonblanchable purpuric rash or petechiae, and bleeding from the nasal passages, genitourinary tract, and gingival surfaces [6]. A platelet count of less than 100,000/μL and a peripheral blood smear showing normal WBC and RBC morphology with no schistocytes are common findings [6-8]. Primary ITP is an acquired thrombocytopenia, whereas secondary ITP is thrombocytopenia triggered by another disorder, such as systemic lupus erythematosus, rheumatoid arthritis, hypothyroidism, HIV, hepatitis C, chronic lymphocytic leukemia, or lymphoma [8]. Primary ITP can be managed with corticosteroids, whereas secondary ITP requires treatment of the underlying condition [8].

ES has been reported to copresent with other autoimmune-related disorders, including systemic lupus erythematosus [9], autoimmune lymphoproliferative syndrome [10], Hashimoto thyroiditis [11], rheumatoid arthritis [12], and autoimmune hepatitis [13]. However, there is only one other documented case of multiple sclerosis (MS) in the setting of ES [14]. MS is an autoimmune disease that results in demyelination of the CNS. This disorder manifests with variable clinical and pathologic presentations, which can be attributed to its polygenic pathogenesis. The etiology of MS remains unknown [15]; however, there are multiple working theories regarding the underlying pathophysiology. Multiple risk factors are associated with the development of MS, including genetic susceptibilities, largely in regulatory immunologic functional regions [16], viral infection, namely Epstein-Barr virus [17], geographic factors [18], and other environmental factors [19].

The original hypothesis regarding the pathophysiology of MS focused on the role of T cells. Autoreactive T cells originate in the periphery; then, through a triggering event, peripheral autoimmune T cells infiltrate the CNS. This induces inflammatory destruction of myelin and axons, leading to demyelination [20]. T helper 17-type (TH17) cells can secrete IL-17; the population of IL-17-positive TH17 cells is significantly elevated in areas of active MS lesions [21]. IL-17-positive TH17 cell types are also implicated in the destruction of blood-brain barrier tight junctions, resulting in their transmigration into the CNS, where their expression of granzyme B and recruitment of CD4+ lymphocytes result in a large CNS inflammatory reaction [22].

B cells are more recently being investigated for their role in the pathogenesis of MS. B cells have previously been demonstrated to have some involvement, with diagnostic criteria for MS including the identification of clonally expanded B cells and oligoclonal immunoglobulin bands in cerebrospinal fluid [23]. Research has shown that B cells can have multiple roles, including the secretion of autoantibodies targeting myelin oligodendrocyte glycoprotein, myelin basic protein, myelin-derived lipids, neurofascin, contactin-2, KIR4.1, and sperm-associated antigen 16 [20]. These autoantibodies can cause inflammatory destruction of the CNS through opsonization and complement fixation. Additionally, B cells facilitate antigen presentation with IL-mediated T-cell modulation, resulting in T-cell-mediated destruction [20].

We examined the case of a female in her mid-40s with a known diagnosis of MS and presumed ITP. However, further diagnostic workup revealed that her diagnosis of ITP was incorrect, and she was subsequently found to have ES. This case of MS in the setting of ES demonstrates an exceedingly rare co-occurrence of these autoimmune disorders and allows for exploration of a possible shared autoimmune dysregulatory pathogenesis.

## Case presentation

A 46-year-old female with a past medical history of MS and ITP presented with new-onset hematuria from her chronic suprapubic catheter. She had multiple hospitalizations for her ITP and was followed closely with hematology. Her current management on admission included eltrombopag 75 mg daily, weekly IVIG infusions for her ITP, and interferon beta for her MS. Her most recent IVIG infusion was administered 1 day prior to admission. She had also completed a four-week course of rituximab prior to this hospital admission.

Upon admission, she was tachypneic and tachycardic, with a urine culture positive for *Pseudomonas aeruginosa *growth. Laboratory testing revealed an elevated lactate level of 4.3 mmol/L, consistent with sepsis secondary to a urinary tract infection. The patient was treated for urosepsis; however, additional laboratory abnormalities raised concern for underlying hematologic disease processes.

**Figure 1:**
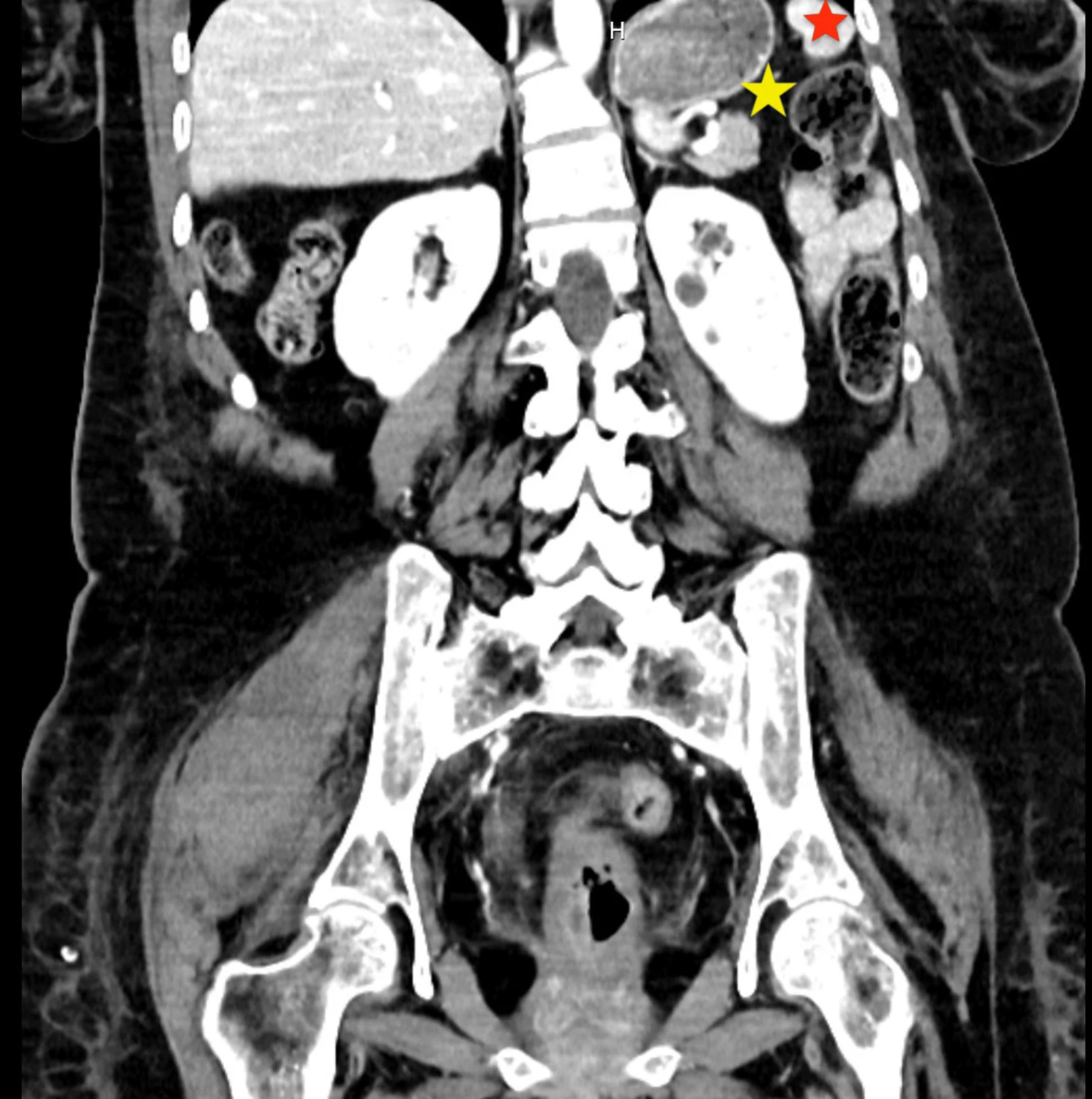
CT abdomen and pelvis with contrast on hospital admission day 1 showing no intra-abdominal hemorrhage or free fluid. The red star indicates the spleen, while the yellow star indicates the retroperitoneal space.

Throughout admission, the patient received four units of packed RBCs and eight units of platelets due to significant anemia and thrombocytopenia. On hospital day 4, the patient received two rounds of IVIG along with pulsed high-dose dexamethasone (40 mg daily for four days) in an attempt to raise her platelet count to greater than 30,000/μL (per hematology) prior to discharge. The patient’s laboratory values (Table 1), further anemia workup (Table 2), and negative CT of the abdomen for intra-abdominal bleeding (Figure 1) confirmed a diagnosis of wAIHA plus ITP, resulting in a new diagnosis of ES. The patient demonstrated a promising response to IVIG and high-dose dexamethasone, allowing for discharge on day 7. Hematology was consulted prior to discharge; fostamatinib, an oral spleen tyrosine kinase (SYK) inhibitor, along with splenectomy, was discussed as future treatment options if rituximab continued to yield little clinical improvement.

**Table 1:** Patient laboratory values.

Parameter	Day 1 (admission)	Day 3	Day 7 (discharge)	Reference	Unit
Hemoglobin	10.8	6.8	7.6	12.0-16.0	g/dL
Hematocrit	33.1	20.1	22.8	36-46	%
WBC	3.9	13.2	12.2	4.5-11.0	× 10³
Platelets	16	4	62	150,000-400,000	mm³

**Table 2:** Anemia workup. DAT, direct antiglobulin test

Parameter	Value	Reference range	Unit
DAT	Positive	Negative	-
IgG	2,493	635-1,741	mg/dL
Reticulocytes	5.1	0.5-1.5	%
Haptoglobin	<30	44-215	mg/dL
Total bilirubin	1.4	0.1-1.0	mg/dL

A previous bone marrow biopsy completed by an outpatient hematologist two months prior to this hospital admission revealed normocellular marrow with maturing trilineage hematopoiesis and slight erythroid predominance.

## Discussion

This case highlights several important aspects of ES, including delayed recognition related to its relapsing course, polyautoimmunity in the setting of MS, and the therapeutic efficacy of rituximab and fostamatinib therapy.

This patient most likely exhibits primary ES, given the absence of identifiable underlying causes. Causes of secondary ES include lymphoproliferative disorders, common variable immunodeficiency, and inborn errors of immunity [24,25]. The patient’s normocellular bone marrow biopsy results further ruled out causes of secondary ES [24]. This patient’s diagnosis of ES was delayed by 17 years, resulting in initial management being limited to ITP. As demonstrated in this case, it is important for physicians and other healthcare providers to consider ES in patients with an ITP diagnosis and recurrent hospitalizations secondary to treatment-resistant thrombocytopenia.

Rituximab is a monoclonal antibody that leads to B-cell depletion by binding to the CD20 antigen on the surface of B lymphocytes, causing immune-mediated destruction. Although an initial response to rituximab can be observed, relapsing and refractory ITP is common. Patients with multirefractory ITP tend to produce a higher proportion of anti-GPIb/IX antibodies, leading to more rapid clearance of platelets [26]. In patients with refractory and recurrent ITP, as in the case discussed above, it is important to consider alternative treatments to prevent significant drops in platelet levels and decrease the risk of future hospitalizations. This patient’s lack of response to rituximab therapy is a point of emphasis, as it is the recommended second-line therapy for ES in the setting of persistent disease, presence of antiphospholipid antibodies, and previous thromboembolic events [24]. However, rituximab failure is a documented phenomenon associated with ES, as well as isolated autoimmune cytopenias. The underlying mechanism of rituximab failure in this patient population can be attributed to T-cell-mediated activity. Studies demonstrate that ITP patients who do not respond to rituximab therapy exhibit preferential splenic CD8+ T-cell activation with TH1 and Tc1 polarization, leading to continued T-cell-mediated platelet destruction despite rituximab-mediated B-cell depletion [27]. This suggests that, in clinical scenarios where rituximab fails to control autoimmune cytopenia, as seen in this patient, T-cell-mediated mechanisms may play a significant role in cytopenia pathogenesis.

The discussion of rituximab failure attributable to T-cell-mediated activity emphasizes another potential point of further investigation: polyautoimmunity. The concept of polyautoimmunity is defined as the presence of two or more autoimmune diseases in a single individual [28]. Polyautoimmunity emphasizes the concept of a possible common origin for co-occurring diseases, suggesting a potential common pathological factor described as autoimmune tautology [29]. This case presents an exceedingly rare example of polyautoimmunity with the coexistence of ES and MS. It further raises the possibility of overlapping immune dysregulation. Although the mechanisms underlying each disease differ, both have increasingly recognized contributions from dysregulated B- and T-cell responses, resulting in aberrant autoimmunity and cell destruction. It is therefore possible that shared pathological B- or T-cell function and impaired upstream immune regulation may predispose certain individuals to dual autoimmunity, resulting in both conditions. Other studies have demonstrated that MS has an increased prevalence of polyautoimmunity compared with the general population, supporting the concept of potential autoimmune tautology [29]. Although no causal relationship has been established from this case, especially in the context of failed rituximab therapy, it highlights an area for further investigation.

The hematology consultation appropriately identified fostamatinib, an oral SYK inhibitor, as a next-line therapy for this patient’s relapsing ES. SYK is expressed in hematopoietic cells and functions as a central mediator of activating Fc receptors and B-cell receptor signaling pathways [30]. This mechanism is particularly relevant in our patient’s case, as rituximab failed to control her cytopenias despite targeting B cells. Rituximab-mediated depletion of CD20-positive B cells, however, does not eliminate long-lived plasma cells or directly inhibit Fc receptor-mediated phagocytosis of antibody-coated blood cells. In contrast, SYK inhibition targets downstream immune effector pathways mediated by macrophage destruction of opsonized erythrocytes and platelets, while also attenuating B-cell receptor signaling. Therefore, fostamatinib may remain effective despite poor clinical response to B-cell depletion alone. The 2024 consensus guidelines recommend fostamatinib for patients with two or more thrombocytopenia relapses, especially those with previous thrombotic events [24], as responses to treatment have been observed as early as 15 days [31].

The discussion of splenectomy in this case appropriately acknowledged its reduced efficacy in ES compared with isolated ITP [32]. However, splenectomy should be reserved for steroid-refractory and fulminant ITP, as it can increase infectious and thrombotic risks when performed in patients with other co-occurring autoimmune diseases [33].

Enrollment in clinical trials for patients with ES who fail standard therapies should be strongly considered. Novel agents currently undergoing trials include newer SYK inhibitors, such as sovleplenib, Bruton tyrosine kinase inhibitors, complement inhibitors, and, in refractory cases, hematopoietic stem cell transplantation [24,34,35]. This case emphasizes the importance of early recognition of ES in patients who present with relapsing autoimmune cytopenias, along with the need for comprehensive workups to exclude secondary causes. As the pathogenesis of MS continues to be explored, current discussions of T- and B-cell dysregulation overlap with the underlying etiology of ES, suggesting a possible shared aberrant pathway. Additionally, consideration of additional treatment options, including fostamatinib, represents an evidence-based approach to clinical decision-making through the utilization of a mechanistically distinct therapy.

## Conclusions

Patients with ES co-occurring with MS require heightened clinical awareness and comprehensive evaluation. The inherent complexity and diagnostic challenges of ES, particularly when it presents alongside other autoimmune conditions, such as MS, can prolong diagnostic delays. Initial misclassification as isolated ITP and subsequent treatment resistance highlight the importance of maintaining a broad differential diagnosis in patients with recurrent cytopenias. This patient’s clinical course demonstrates the limitations of standard therapies such as corticosteroids, IVIG, and rituximab and reinforces the need to consider mechanistically distinct agents, such as fostamatinib, when B-cell-depleting treatments fail to achieve durable responses. SYK inhibitors, such as fostamatinib, have demonstrated efficacy and should be explored further. There is potential for a shared autoimmune pathogenesis between ES and MS, which requires further investigation. Overall, this case illustrates the value of early recognition, careful exclusion of secondary causes, and thoughtful escalation of therapy.
